# Perceived Stigma and Its Association with Gender and Disclosure Status among People Living with HIV/AIDS and Attending Antiretroviral Therapy Clinics in Ethiopia: A Systematic Review and Meta-Analysis

**DOI:** 10.1155/2022/3246249

**Published:** 2022-07-08

**Authors:** Chalachew Kassaw, Daniel Sisay, Ephrem Awulachew, Habtamu Endashaw Hareru

**Affiliations:** ^1^Department of Psychiatry, College of Health and Medical Science, Dilla University, Dilla, Ethiopia; ^2^School of Public Health, College of Medicine and Health Science, Dilla University, Dilla, Ethiopia; ^3^Department of Medical Laboratory Science, College of Health Science and Medicine, Dilla University, Dilla, Ethiopia

## Abstract

**Background:**

The psychological experience of being rejected, blamed, and ashamed in relation to a recognized medical disease is known as perceived stigma. It has a close connection to psychological health and therapy afterward. To the best of our knowledge, there has not been any national systematic review and meta-analysis research on this topic. Therefore, we conducted this analysis to thoroughly evaluate the pooled prevalence of perceived stigma among HIV/AIDS patients in Ethiopia who are receiving antiretroviral therapy and its relationship to gender differences and disclosure status.

**Method:**

We investigated the eight databases for quantitative Ethiopian studies published in English from 2008 to 2021 that looked at the relationship between felt stigma, gender, and disclosure status. To meet the statistical requirements of a systematic review and meta-analysis analysis, the random effect model for pooled prevalence of perceived stigma, log odds ratio for associated variables, *I*-squared statistics for heterogeneity, and Egger's test for publication bias were implemented. The Joanna Briggs Institute Meta-Analysis of Statistics Assessment and Review Instrument's standard data extraction method was performed to collect the necessary data, and STATA-14 statistical software was used for analysis.

**Result:**

A total of 8 cross-sectional Ethiopian studies with 3,857 participants were integrated into this systematic review and meta-analysis study. The pooled prevalence of perceived stigma among people living with HIV/AIDS and attending antiretroviral therapy in Ethiopia was OR = 50.36% (95% CI: (40.71, 60.00), *I*^2^ = 97.3%, *p*=0.000 ). The pooled odds ratio of being male was 0.95 (95% CI: 0.53, 1.68, *I*^2^ = 86.7%, *p*=0.000) and disclosure status was 0.84 (95% CI: 0.09, 7.89, *I*^2^ = 97.9%, *p*=0.000).

**Conclusion:**

In this study, half of the participants encountered stigma. There was no statistically significant correlation between gender difference, disclosure status, and the perception of stigma. To address the mental and psychological issues of people living with HIV/AIDS, it is necessary to look into other factors that influence perceived stigma. It is recommended to screen for and treat perceived stigma with prompt examination and follow-up.

## 1. Background

When someone feels stigma, it is because of a particular circumstance or value that makes them stand out from other members of society. It is a personal sense of shame, abandonment, and prejudice [1]. Globally, HIV/AIDS affected around 36 million people and remains a nonemergency public health issue. Despite the fact that HIV now affected every country on the Earth, it has spread to epidemic proportions over most of Africa [2]. In Sub-Saharan Africa, there are about 34 million living with HIV/AIDS [3]. Women are disproportionately affected and accounted for 58% of the total HIV-positive people living in the region. Sub-Saharan Africa accounted for 71% of all AIDS-related deaths worldwide in 2011 [4].

Discrimination based on natural identities, such as gender, skin color, physical illness, such as HIV/AIDS, epilepsy, and mental illness, is the outcome of unreasonable or negative attitudes, behaviors, and judgments [5–8]. Self-esteem, making and keeping relationships, getting a job, and readiness to expose mental illness were highly related to perceived stigma [9, 10]. The prevalence of perceived stigma among people living with HIV/AIDS in Ethiopia was ranging from 28.9% [11] to 72.2% [12]. Females are more sensitive to the medical and social effects of HIV/AIDS, such as facing more unfavorable reactions in society than their male counterparts [13].

Self-doubt, low self-esteem, feelings of prejudice, and loneliness result from disclosing one's HIV/AIDS status to others. Nonetheless, it is the foundation for getting medical therapy, care, and psychological support, as well as avoiding self-blaming and nonadherence and promoting improved health [14]. A cross-sectional study conducted in northern and southern Ethiopia [11, 15] showed that those patients of female gender had two times more likely to develop perceived stigma than males. However, other cross-sectional studies done in the Oromia region [16] and Southwest Ethiopia [17] showed that there was no statistical difference between females and males for experiencing perceived stigma due to seropositive status.

To maintain psychological health, a person must be aware of the underlying causes, progression, and treatments of their condition, as well as the effects on their life, the symptoms that are related to it, and how they can manage or treat it [18].

It is well known that psychological stress's biological regulators and pathways can lead to mood disorders, change the way the central immune system responds, and eventually affect the onset and course of viral-induced illnesses like the flu, herpes, or HIV [19].

Less perceived stigma, better social relationships, health, physical well-being, coping mechanisms, prosocial activity involvement, problem-solving abilities, and creativity across multiple life domains are associated with patients who reveal their seropositive status to others [20]. Patients who have a high dispositional hope level are highly motivated and can foresee alternatives when faced with obstacles, and they may think that the present circumstance and obstacles can be managed and overcome [21].

To address the overall health impact of the patient's condition, it is essential to address the elements that are likely to contribute to perceived stigma secondary to HIV/AIDS status. The relationship between perceived stigma, gender, and disclosure status has been an issue of debate, but no national systematic review and meta-analysis research has been published on the subject. Additionally, no systematic review of this subject is in progress or planned, according to a preliminary search of our database. Therefore, the purpose of this study was to assess perceived stigma and its relationship with gender and disclosure status among people living with HIV/AIDS and attending Antiretroviral Therapy Clinics in Ethiopia.

## 2. Methods and Materials

### 2.1. Search Strategy

To identify eligible literature for the current systematic study, eight electronic databases such as Scopus, HINARI, PubMed, Google Scholar, Google Science Direct, Web of Science, World Health Organization African Index, and Cochrane electronic were used as search engines. In addition, manual search methods have been carried out to find peer-reviewed literature on the magnitude of perceived stigma and its association with gender and disclosure status among people living with HIV/AIDS and attending ART treatment. The search was carried out from August 25, 2021, to Nov 10, 2021, and articles included in the review were the literature published from 2008 to June 2021.

The search strategy was employed using the following keywords (magnitude) OR (prevalence) OR (proportion) OR (incidence) AND (perceived stigma) AND (Human immunodeficiency virus) OR (“HIV” Infections) OR (AIDS) OR (Acquired immunodeficiency syndrome) AND (antiretroviral therapy) OR (ART) OR (highly active antiretroviral therapy) OR (HAART) AND (associated factors) OR (risk factors) OR (predisposing factors) AND (gender difference) OR (disclosure status) AND Ethiopia(Supplementary 1). This review was designed as per the identified characteristics from reports on the Preferred Reporting Items for Systematic Reviews and Meta-Analyses (PRISMA) (Supplementary 2):

  Eligibility criteria.
  Inclusion criteria.
  Study area: Ethiopian studies
  Population: participants aged ≥18 years old, living with HIV/AIDS and attending antiretroviral therapy (ART).
  Exposure: determinants of perceived stigma. Gender and disclosure status are variables associated with the outcome variable.
  Publication condition: only published studies.
  Study design: all observational studies (cross-sectional, cohort, and case-control) report the prevalence of perceived stigma and its association with gender and disclosure status.
  Language: only articles reported in English language.
  Outcome: perceived stigma among people living with HIV/AIDS and attending ART clinics.

### 2.2. Exclusion Criteria

The studies that did not present the outcome variable, published studies that were not accessible after at least two email contacts with the corresponding author, qualitative studies, meta-analysis, and systematic reviews were excluded.

### 2.3. Outcome Measurements

This review measured two objectives—the first objective was to determine the pooled prevalence of perceived stigma among people living with HIV/AIDS and attending antiretroviral therapy in Ethiopia which was determined by dividing the number of participants who had perceived stigma by the total number of participants included in the study (sample size) multiplied by 100. The second objective was to assess the association between perceived stigma with gender and disclosure status.

### 2.4. Data Extraction

Four study authors (CK, HE, EA, and DS) were independently screened and extracted all the necessary data by using a standardized data extraction tool of Joanna Briggs Institute Meta-Analysis of Statistics Assessment and Review Instrument (Supplementary 3). All extracted data were filled on a prepared Microsoft Excel sheet. Any disagreement among the four reviewers in the study selection, validity assessment, and data extraction was resolved by consensus with all reviewers through discussion and double extraction of the inconsistent data together. The data extraction format includes the first author's name, year of publication, study region, study period, sample size, and the instrument used to measure the perceived stigma and its association with gender difference and disclosure status.

### 2.5. Quality Appraisal

The Joanna Briggs Institute Critical Appraisal instruments, which have 9 questions with yes, no, unclear, and not relevant answers, were used to assess the methodological quality of the studies [22] (Supplementary 4). The risk of bias was determined using the Agency for Healthcare Research and Quality (AHRQ) standards, and the corresponding authors of each study were contacted to clarify any missing or unclear data [23] (Supplementary 5).

### 2.6. Quality Assessment

All authors were assessed independently the qualities of the original articles by using the Newcastle–Ottawa Scale quality assessment tool for cross-sectional studies [24]. The assessment tool consists of three main segments: the first segment assesses the methodological quality of each study; the second section inspects the comparability of the studies; and the last segment measures statistical analysis and the outcome of the study article.

### 2.7. Data Analysis

The data were extracted using a Microsoft Excel spreadsheet and exported to STATA version-14 statistical software for analysis. Heterogeneity between the included articles was checked by heterogeneity *χ*2 test and *I*^2^ test [25]. Publication bias was examined by performing Egger's correlation and Begg's regression intercept tests at a 5% significant level [26]. Subgroup analysis was conducted based on the region of studies, publication year, and sample size to minimize the random variations between the point estimates of the primary studies. The point estimates with their 95% confidence interval were presented using texts, table, and forest plots. Pooled odds ratio at a 95% confidence interval was calculated to determine the association between perceived stigma, gender difference, and disclosure status. Small studies and deviant results from the rest of the studies were omitted, and small studies and deviant results from the rest of the studies were entered into a sensitivity test.

## 3. Result

### 3.1. Identification of Study

**Figure 1:**
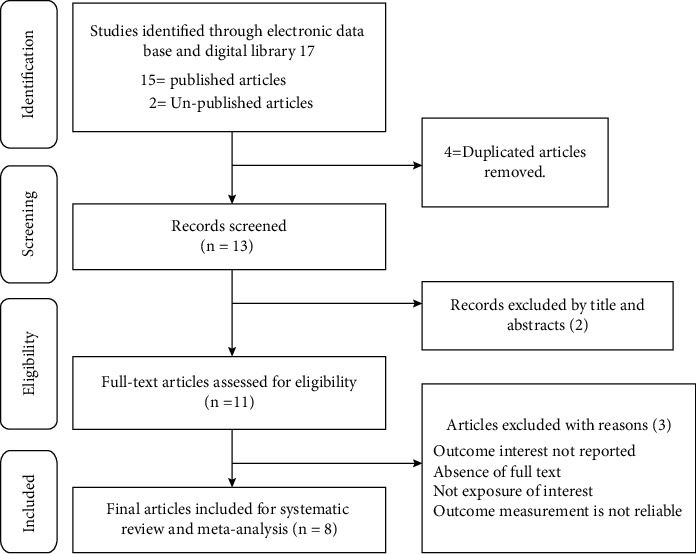
Flow diagram showing the findings of the systematic search and the criteria for exclusion.

A total number of 29 articles were recruited through electronic databases (Google Scholar, PubMed, Cochrane Library, Web of Science, and Google Science Direct) and digital library searches; after removing duplicates, a total number of 15 items were retrieved of which 4 items were rejected just by reading the titles and abstracts. And, full-text copies of the remaining 13 articles that met, or potentially met, the inclusion criteria were assessed. After further screening, 8 papers were included for analysis (Figure 1).

### 3.2. Characteristics of Included Studies

A total of 8 cross-sectional studies were conducted from 2016 to 2021 G.C. with 3, 857 participants were included in this systematic review and meta-analysis study. All incorporated studies were institution-based cross-sectional studies fulfilling the predefined inclusion criteria. Out of these included studies, two were from the Amhara region [15, 27], four studies were from the Oromia region [12, 17, 28, 29], and the last two studies were from the southern part of the country [11, 30]. Four of the eight studies included in the meta-analysis utilized an HIV stigma psychometric assessment scale for the assessment of perceived stigma. The size of the sample utilized by the studies ranged from 270 to 1175 participants, and half of the studies used a systematic random sampling technique to select their study participants (Table 1).

### 3.3. Quality of Included Studies

In this systematic review, all of the included studies were measured using the Newcastle–Ottawa quality assessment scale [24]. The scoring difference among authors was solved by taking the mean score of their assessment results. Overall, the average score was 8 which ranges from 6 to 9. Amongst all studies, two of the studies scored above the average score for quality assessment, and the rest of six studies were scored above the average score (Supplementary 6).

### 3.4. Prevalence of Perceived Stigma

**Figure 2:**
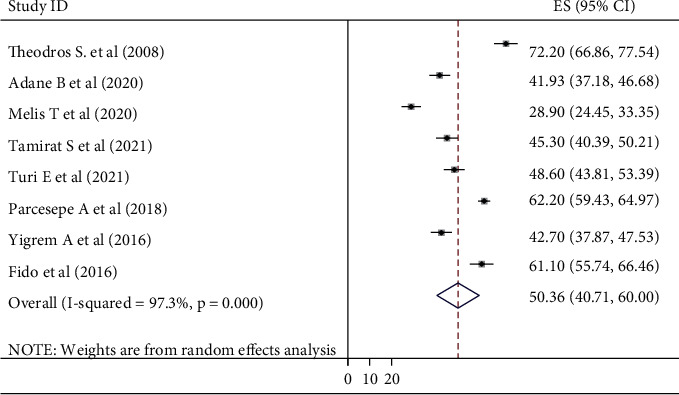
Pooled prevalence of perceived stigma among people living with HIV/AIDS and attending antiretroviral therapy in Ethiopia, 2021.

The pooled prevalence of perceived among people living with HIV/AIDS and attending ART treatment from eight included studies conducted in Ethiopia was 50.36 ([40.71, 60.00]) with significant heterogeneity among the studies (*I*^2^ = 97.3%, *p*=0.000) (Figure 2).

### 3.5. Publication Bias

**Figure 3:**
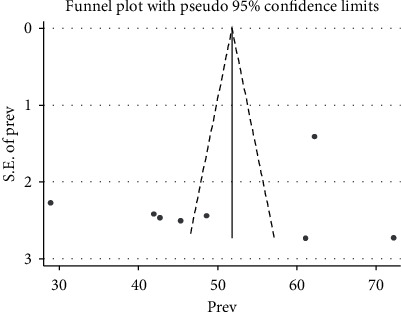
Funnel plot graph on the pooled prevalence of perceived stigma among people living with HIV/AIDS and attending antiretroviral therapy in Ethiopia, 2021.

Regarding publication bias, the funnel plot was visually inspected for symmetry; there was an asymmetric distribution of the effect estimates. To confirm this, Begg's and Eggers' tests were checked and no significant publication bias was observed as evidenced by *p*=0.386 and*p*=0.424, respectively (Figure 3).

### 3.6. Subgroup Analysis

This systematic review performed a subgroup analysis based on the regions, publication year, and sampling technique to assess the pooled prevalence of perceived stigma. Out of the three listed regions, the lowest prevalence of perceived stigma (35.76 (22.243–49.29)) was found in the Southern Nations, Nationalities, and Peoples' Region (SNNPR), whereas the highest (60.98 (52.622–69.35)) was from the Oromia region. Another subgroup analysis was the sampling technique, the pooled prevalence of perceived stigma was higher among studies that used nonrandom sampling (52.658 (34.011–71.305)). The last subgroup analysis was publication year. The pooled prevalence of perceived stigma among studies conducted before 2018 was 59.534 (48.752–70.31) which was higher compared to the studies conducted after 2018 G.C. (Table 2).

### 3.7. The Association between Perceived Stigma and Gender Difference

**Figure 4:**
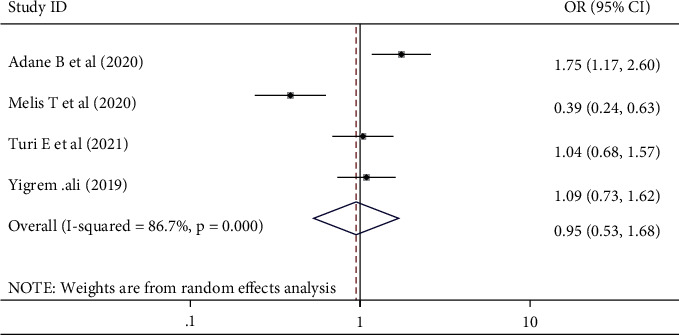
The pooled odds ratio showed the association between perceived stigma and gender difference among people living with HIV/AIDS and attending ART in Ethiopia, 2021.

A significant association between perceived stigma and the female gender was described in the results of four studies [11, 15, 28, 31]. The current review result showed that the pooled odds ratio of being male was 0.95 (0.53, 1.68) times to experience perceived stigma with a random effect model (*I*-squared = 86.7%, *p*=0.000) (Figure 4).

### 3.8. The Association between Perceived Stigma and Disclosure Status

**Figure 5:**
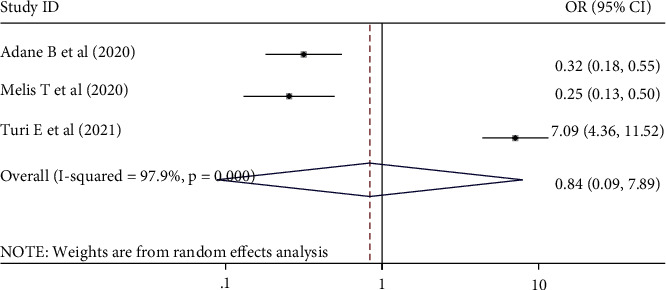
The pooled odds ratio showed the association between perceived stigma and disclosure status among people living with HIV/AIDS and attending ART in Ethiopia, 2021.

The association between perceived stigma and disclosure status was also shown in the results of the three studies [11, 15, 28]. The current review result indicated that the pooled odds ratio of disclosed their seropositive status for others was 0.84 (0.09, 7.89) times to develop perceived stigma with a random effect model to estimate the associations was overall (*I*-squared = 97.9%, *p*=0.000) (Figure 5).

## 4. Discussion

In this systematic review and meta-analysis study, eight studies with a total of 3, 857 participants were included to estimate the best available evidence for the pooled prevalence of perceived stigma and its association with gender and disclosure status among people living with HIV/AIDS and attending ART in Ethiopia.

The current systematic review and –meta-analysis results showed that the estimated pooled prevalence of perceived stigma among people living with HIV/AIDS and attending ART in Ethiopia was 50.36 [40.71, 60.00]. This study finding was inconsistent with the country-level preliminary study conducted in South Africa by 43% [32]. However, this study's finding was lower as compared to the systematic review and meta-analysis study conducted in low-income countries by 82% [33]. The possible explanation for this difference might be the previous study which was conducted before 7 years, and activities like the creation of anonymous groups, the integration of mental health services, and the accessibility of medication have all made good progress over time, which helps to resolve such self-blaming, discriminatory thoughts, feelings, and behavior patterns. Moreover, it might be attributed to the sociocultural and study period variances.

This study found that there is no statically significant association between perceived stigma and gender among people living with HIV/AIDS and attending ART treatment which was a similar finding to a systematic review carried out in North America [34] and a contradictory result with a primary preliminary study conducted in South Africa [32] in which females were more likely to develop a perceived stigma. This study's finding was also different compared to the study conducted in India in which males had high HIV-related stigma score than females [35, 36]. Due to its seropositive status, gender difference may not have a biological basis for stigma, but instead is strongly correlated with society's values, attitudes, and ideas regarding male or female gender.

Another associated factor related to perceived stigma was disclosure status in which this study found that there is no statically significant association between perceived stigma and disclosure status. This study finding was in line with the primary study conducted in the western province of South Africa [37] and a systematic review study conducted in North America [34]. However, this finding was contrary to the meta-analysis study conducted in the United States [38] which showed that patients with less disclosure status had more HIV-related stigma.

It could be explained by the relationship between disclosure status and differences in societal perception and awareness of the illness. Additionally, the patient's diagnostic period, which is closely associated with the role shift, developing coping mechanisms, and joining support networks, all of which serve as pillars for avoiding isolation, placing blame, and being assertive.

### 4.1. Limitation of the Study

The entire paper under consideration was cross-sectional, with small sample sizes, which could have an impact on the pooled estimate. One of the review's potential limitations was the inclusion of a small number of researchers, which could increase standard errors, and the examined studies were only from three regions of Ethiopia, which may not reflect the country's total population.

## 5. Conclusions

In this study, stigma was experienced by nearly half of the participants. Overall, it was shown that stigma perception was not significantly correlated with gender or disclosure status. It would therefore be preferable if pertinent stakeholders, researchers, and volunteers who work with HIV/AIDS patients conduct a qualitative study to identify other factors that influence the emergence of perceived stigma. A long-term plan for screening and counseling sessions should also be developed by mental health professionals working in ART clinics.

## Figures and Tables

**Table 1:** The characteristics studies are included in the current systematic review and meta-analysis.

Author	Year	Region	Assessment tool	ST	N	population	Prevalence of perceived stigma (%)	Key findings	Mean (±SD)	
Theodros S. et al.	2008	OROMIA	HIV stigma psychometric assessment scale	CS	270	HIV+ Adults	72.2	Gender and disclosing HIV-positive status were not significantly associated with perceived stigma.	72.20 (%)	61.1
Adane B et al.	2020	AHMARA	40-point HIV stigma psychometric assessment scale	SS	415	HIV+ Adults	41.93	Stigma was associated with female gender (AOR = 2.08, 95% CI: (1.26, 3.46)), and not disclosing HIV status (AOR = 2.36, 95% CI: (1.19, 4.66)).	41.93	
Melis T et al.	2020	SNNPR	HIV stigma psychometric assessment scale	CS	399	HIV+ Adults	28.8	Stigma was associated with female gender being a female (AOR: 2.5; 95% CI: (1.41,4.12)) and disclosing HIV positive status (AOR: 6; 95% CI: (2.3, 14.9))	28.80	
Tamirat S et al.	2021	AHMARA	HIV-stigma scale	SS	395	HIV+ Adults	45.3	Gender and disclosure status were not significantly associated with perceived stigma	45.30	
Turi E et al.	2021	OROMIA	HIV-stigma scale	CS	418	HIV+ Adults	48.6	Stigma was associated with female sex (AOR = 2.10, 95% CI 1.15–3.82) and nondisclosure of HIV status (AOR = 2.00, 95% CI: 1.11–3.59)	48.60	
Parcesepe A et al.	2018	OROMIA	HIV-related stigma	SS	1175	HIV+ Adults	62.2	Gender and disclosure status were not significantly associated with perceived stigma	62.20	
Yigrem A et al.	2016	SNNPR	HIV stigma scale	SS	403	HIV+ Adults	42.7	Stigma was associated with Females (AOR = 2.4, 95% CI: 1.28–4.33) and disclosure status was not significantly associated with perceived stigma	42.70	
Fido et al.	2016	OROMIA	HIV stigma scale	CS	318	HIV+ Adults	61.1	Female gender (*β* = 6.73 (3.3–10.2)) and disclosure status were not significantly associated with perceived stigma	61.10	

N.B: ST; sampling technique, CS; convenient sampling, SS; systematic sampling.

**Table 2:** Subgroup analysis result of the study.

Variables	Characteristics	Included studies	Prevalence rate (95% CI)
Region	Amhara Oromia SNNPR	4 4 2	43.55 (40.146–46.97) 60.98 (52.622–69.35) 35.76 (22.243–49.29)
Publication year	≤2018 G.C. ≥2018 G.C.	4 4	59.534(48.752–70.31) 41.147(32.412–49.88)
Sampling technique	Random (systematic) Nonrandom (convenient)	4 4	48.138(36.679–59.59) 52.658(34.011–71.305)
Overall		8	50.356 (40.712–60.00)

## Data Availability

The datasets used and/or analyzed during the current study are included in Supplementary Materials.

## Abbreviations

ART: Antiretroviral therapy
CI: Confidence interval
WHO: World Health Organization
UNAIDS: United Nations Programme on HIV/AIDS
PLWHA: People living with HIV/AIDS
SNNP: Southern Nation and Nationalities of Ethiopia
PRISMA: Preferred Reporting Items for Systematic Reviews and Meta-Analyses
STATA: Data analysis and statistical software
PICO: Population, Intervention, Comparator, and Outcome
OD: Odds ratio.

## Supplementary Materials

Supplementary Materials Supplementary 1: full searching strategy by databases. Supplementary 2: the PRISMA 2020 statement: an updated guideline for reporting systematic reviews. Supplementary 3: data extraction instruments. Supplementary 4: appraisal instruments. Supplementary 5: risk of bias assessment within the studies. Supplementary 6: quality assessment of the study.
